# Molecular dynamics simulation-based study to analyse the properties of entrapped water between gold and graphene 2D interfaces†

**DOI:** 10.1039/d3na00878a

**Published:** 2024-03-21

**Authors:** Shashank Mishra, Fengyuan Liu, Dhayalan Shakthivel, Beena Rai, Vihar Georgiev

**Affiliations:** a James Watt School of Engineering, University of Glasgow G12 8QQ Glasgow UK shashank.mishra@glasgow.ac.uk; b TCS Research, Tata Consultancy Services Limited Pune 411013 India

## Abstract

Heterostructures based on graphene and other 2D materials have received significant attention in recent years. However, it is challenging to fabricate them with an ultra-clean interface due to unwanted foreign molecules, which usually get introduced during their transfer to a desired substrate. Clean nanofabrication is critical for the utilization of these materials in 2D nanoelectronics devices and circuits, and therefore, it is important to understand the influence of the “non-ideal” interface. Inspired by the wet-transfer process of the CVD-grown graphene, herein, we present an atomistic simulation of the graphene–Au interface, where water molecules often get trapped during the transfer process. By using molecular dynamics (MD) simulations, we investigated the structural variations of the trapped water and the traction–separation curve derived from the graphene–Au interface at 300 K. We observed the formation of an ice-like structure with square-ice patterns when the thickness of the water film was <5 Å. This could cause undesirable strain in the graphene layer and hence affect the performance of devices developed from it. We also observed that at higher thicknesses the water film is predominantly present in the liquid state. The traction separation curve showed that the adhesion of graphene is better in the presence of an ice-like structure. This study explains the behaviour of water confined at the nanoscale region and advances our understanding of the graphene–Au interface in 2D nanoelectronics devices and circuits.

## Abstract 2

Heterostructures based on graphene and other 2D materials have received significant attention in recent years.

## Graphical abstract

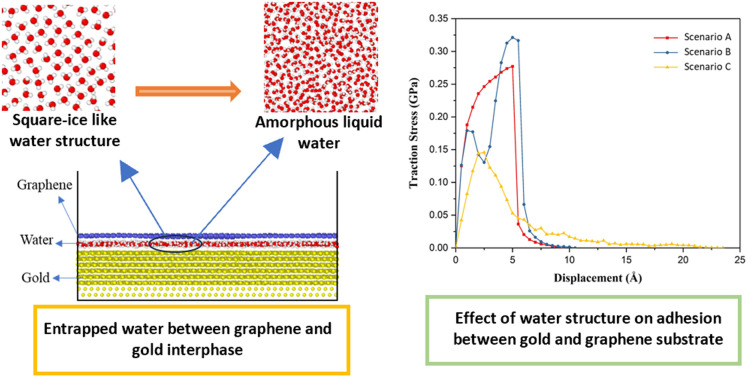

## Introduction

Graphene and other 2D materials have received significant attention in recent years owing to their excellent electrical, optical and mechanical properties, which are attractive for various types of sensors required in energy and electronics devices such as wearables, electronic skin, robotics, and transparent electronics.^1–4^ In particular, the van der Waals (vdW) heterostructures based on 2D materials have opened new opportunities for electronic devices with exceptional electronic and structural properties.^5–9^ Specifically, the large-scale fabrication and synthesis of 2D materials using the CVD method and their transfer onto foreign substrate have been extensively studied in the past two decades, leading to various novel devices such as field effect transistors, photodetection, microelectromechanical systems (MEMS) and flexible sensors.^8,10,11^ However, the realised large-scale vdW structures using the CVD-grown material are normally non-ideal as they come with unintended foreign species (*e.g.*, water) trapped at the interface. These foreign species could have a considerable impact on the uniformity of the device response and hence on the large-scale implementation. Therefore, it is important to understand the widely observed non-ideal interface properties.

Various transfer mediums, including water, alcohol, isopropanol, and heptane, have been used to assist the wet transfer of the 2D materials owing to the capillary force.^12–19^ Among them, water is employed at all the fabrication stages (transfer, patterning and metallization) as a medium, and therefore, understanding the graphene–metal (Au) structure with trapped water has a profound meaning both for research and applications. For example, the graphene–Au interface has considerable influence over the performance of Graphene-based Field Effect Transistors (GFETs) as it defines the contact resistance and hence the efficient charge transfer. Previous studies suggest that water clusters in the range of 0.3–0.5 nm in height and 10–40 nm in width can be found between the gold (111) electrode and graphene.^20^ The trapped water clusters could alter the electronic properties of graphene as well as the device by inducing strong and highly localised electron doping.^20–22^ Additionally, the interface with Au is relevant for many other 2D materials. As an example, in the last years, mechanical exfoliation of transition metal dichalcogenides (TMDs) and other 2D materials on gold surfaces has been investigated by several research groups, and the method proved to be effective in achieving the exfoliation of large-area membranes by exploiting the interaction with Au, as in the case of MoS_2_.^23–26^ Interestingly, a certain variability in the morphological, vibrational and optical properties of exfoliated TMDs has been found by different authors, and this was ascribed to the differences in the gold surface morphology and the presence of hydrocarbon contaminants at the interface. The role played by trapped water at the 2D materials/Au interface on the interaction strength has not been discussed so far. The presence of entrapped water molecules could also alter the adhesion energy,^16^ which plays a crucial role in the fabrication and integration of graphene-based devices.^19^

The confined water at the nanometre scale exhibits anomalous phase behaviour^27,28^ and because of the restricted molecular degrees of freedom, hydrogen bonding and high pressure in nanoconfinement, many unusual structural features have been observed.^29,30^ For example, multi-layered two-dimensional ice has been found even at room temperature and high-pressure conditions.^31^ Square ice, a rare form of ice, has been observed experimentally between graphene sheets even at ambient conditions.^32^ The formation of square ice indicates ∼1 GPa pressure within the confined space. Nanoconfined water has potential applications in molecular electronics as a nanoscale switch,^27^ capacitive sensing,^33^ and in the desalination process.^34^ These observations have led researchers to use computational techniques such as density functional theory (DFT) and molecular dynamics (MD) simulations to understand and analyse the properties of water under nanoconfinement conditions.^28–30,32,35,36^ However, most of these studies are limited to confinement between carbon-based nanostructures such as graphene and carbon nanotubes. Likewise, these simulation techniques have been separately used to study the adhesion and friction between the graphene and gold substrate for ambient dry conditions.^18,37–42^ However, these studies do not consider the role of adsorbed water molecules on the adhesion properties of graphene over the metallic substrate, which has implications for the performance of GFETs. It is important to study the effect of confined water molecules on the gold–graphene adhesion together as different phases of water in a nanoconfined region face the hydrophobic (graphene) and hydrophilic (gold) surfaces at the same time.

In this study, we used MD simulations to investigate the structure of water confined between gold and graphene at a sub-nanometer length scale. The range of water cluster sizes (∼1200–3200 molecules) studied here corresponds to the experimentally observed cluster size at the graphene/Au interface. Different phases of water structure were predicted by varying the thickness of the water film in the simulations. The displayed phase changes (liquid to disordered with a square-ice-like pattern) were sensitive to the narrow difference in water cluster sizes. The traction–separation properties between graphene and gold were also investigated in the presence of adsorbed confined water clusters to understand the variation in adhesion behaviour at different thicknesses of the water films. We observed the formation of an ice-like structure with square-ice patterns when the thickness of the water film is <5 Å. This could cause undesirable strain in the graphene layer and hence affect the performance of devices developed from it. We also observed that at higher thicknesses the water film is predominantly present in the liquid state. This study will help to develop a better understanding of the effect of aqueous nanofabrication conditions on the properties of the nano-scale devices especially the ones involving wet-transferred graphene.

## Results and discussion

**Fig. 1:**
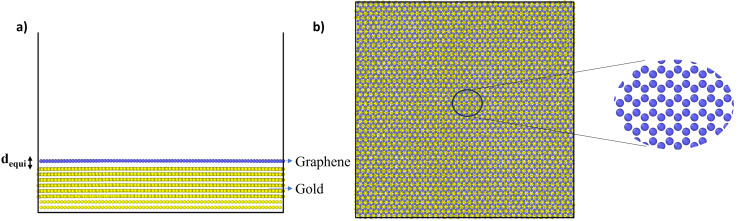
(a) Side and (b) top view of the simulated gold–graphene system with a magnified view of graphene atoms.

In the MD simulations, the interaction between C atoms of graphene and Au atoms of gold substrate were modelled using 6, 12 Lennard-Jones (LJ) interactions with parameters *σ* = 3.42 Å and *ε* = 8 meV, which have been used previously.^38^ With these parameters, and after energy minimization at 0 K, the equilibrium distance (*d*_equi_) and binding energy between gold and graphene were found to be 3.36 Å and 60.7 meV per C atoms, respectively (Fig. 1). When compared with previous DFT studies wherein different pseudopotentials were used to compute *d*_equi_ and binding energy in the range of 3.22–3.52 Å and 30–72 meV per C atoms, respectively, the LJ parameters used in this simulation are consistent with the predictions for these two parameters.^41,42^ This validates the LJ potential parameters used here for C and Au atoms. After energy minimisation at 0 K, the above gold–graphene system was subjected to equilibration in canonical (NVT) ensemble at 300 K. The distance *d*_equi_ increased marginally to 3.42 Å due to the thermal effect on the atoms.

To study the effect of water, confined at the gold–graphene interface, on *d*_equi_ and their structural properties, we considered three different scenarios at varying numbers of water molecules (Table 1). The distances, *d*_equi_ and *d*_thick_ (average thickness of the water film), for all these 3 scenarios (after NVT equilibration at 300 K) are given in Table 1. It can be observed that *d*_equi_ and *d*_thick_ increase upon increasing the number of water molecules. The predicted range of *d*_thick_ lies within 3.5–9.21 Å, *i.e.*, within 1 nm range, which is also generally found in the experimental conditions.^20^ The effect of varying thickness with a number of water molecules on the structural properties of the confined water between gold and graphene as well as the effect on traction–separation properties of graphene is discussed below.

**Table 1:** Number of water molecules, the equilibrium distance between gold and graphene, and the thickness of the water film

Scenario	Number of water molecules	Avg. equilibrium distance between gold and graphene, *d*_equi_ (Å)	Avg. thickness of water film, *d*_thick_ (Å)
A	1200	6.92	3.50
B	1623	8.06	4.64
C	3139	12.63	9.21

**Fig. 2:**
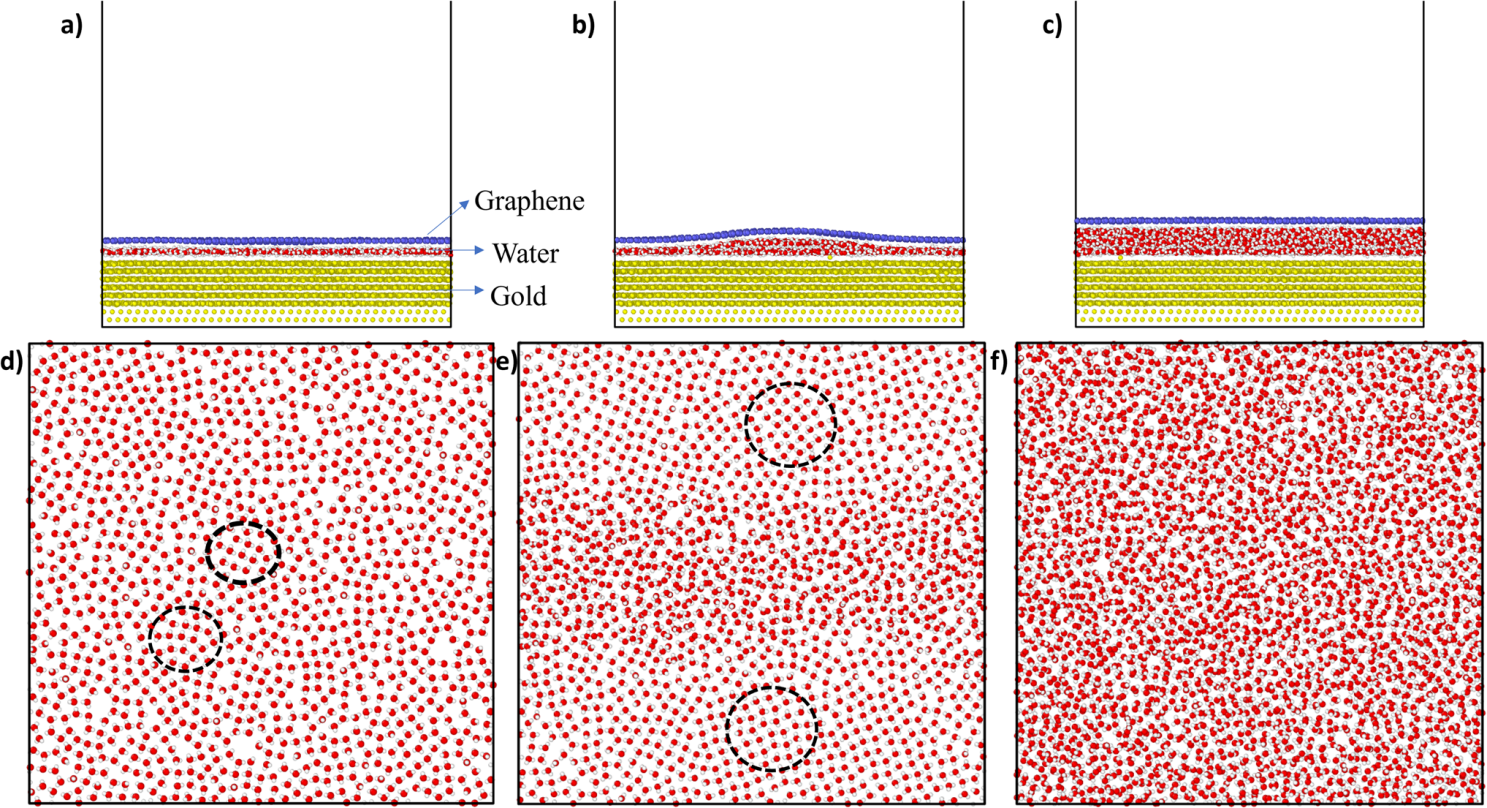
(a), (b), and (c) are the side view snapshots for scenarios A, B, and C, respectively, after NVT equilibration. (d)–(f) depict the structure of water viewed from the top in each of the scenarios after equilibration. The black dotted circles in (d) and (e) show the square-ice-like patterns in the water structure. In all the figures, the red and white atoms correspond to the O and H atoms of the water molecule while blue and yellow atoms correspond to the C and Au atoms of graphene and gold, respectively.

Fig. 2 shows the snapshots of the gold–water–graphene for three scenarios as well as the MD simulated structure of water molecules after the NVT equilibration for each of these scenarios. In scenario A (Fig. 2(a)), which has the least number of water molecules, we observed the formation of a monolayer water film of 3.50 Å thickness (Table 1) with patches of ice-like structure (Fig. 1(d)). The thickness of this monolayer water film is similar to the thickness of the ice-like monolayer water film (3.7 ± 0.2 Å) formed between graphene–graphene and graphene–mica confined surfaces.^43,44^ While in the case of scenario B, we observed a monolayer water film near the edges and a bilayer structure at the centre, potentially inducing a wrinkle in the graphene sheet (Fig. 2b and d).^27,36,45^ Such wrinkles in graphene have also been found in previous experimental studies,^46,47^ and therefore observed results confirm that the efficacy of presented simulations. In the case of scenario C, we observed a completely amorphous water film with a higher thickness between gold and graphene, as seen in Fig. 2c and f. Unlike scenario B, in this case, graphene has a planar structure.

Further analysis of the structure of the water film in scenario A revealed a disordered structure with a square ice-like pattern at some regions (in black dotted circles) as well as some pentagonal and hexagonal rings of oxygen atoms (as shown in Fig. 2(d)). In scenario B, the bilayer structure of water was sliced into two parts: bottom layer and upper layer as seen in the supporting Fig. 1. In the bottom layer, the monolayer water film near the edges has an ice-like structure. However, the ice structure observed here is more ordered near the edges (having a square ice-like pattern) when compared to the structure in the centre or middle of the box. In the upper layer too, the structure is mostly disordered with some regions of square-ice-like patterns. In scenario C, the structure of water appeared to be disordered or amorphous. A layered structure (monolayer, bilayer, or even trilayer) of water is typically observed when it is confined between two hydrophobic materials (*i.e.*, two graphene sheets).^27,36,45^ However, in this study, the water is confined between a strong hydrophobic surface (*i.e.*, graphene) and a weakly hydrophilic surface (*i.e.*, gold), and thus, at sub-nanometer thickness, the confined water transforms from layered ordered ice-like structure (thickness ≤ 5 Å) to amorphous structure (thickness > 5 Å). A similar, transition from an ice-like structure to a liquid structure was observed for water confined between graphene (hydrophobic) and mica (hydrophilic) surfaces.^44^

**Fig. 3:**
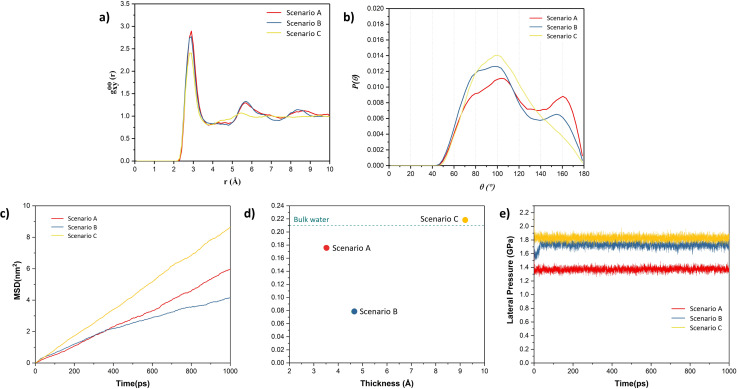
(a) Lateral O–O radial distribution function and (b) O–O–O angle distribution curve in water after NVT equilibration in each of the scenarios A, B and C; (c) lateral MSD with time curve, (d) self-diffusion coefficient *versus* thickness of water films, (e) lateral pressure in the direction parallel to the surface of the graphene sheet *versus* time.

To further validate the presence of ice-like structure in scenarios A and B and amorphous structure in scenario C, the radial distribution curve between O–O atoms and angle distribution between O–O–O atoms were plotted, as shown in Fig. 3. From Fig. 3(a and b), we observed peaks in the radial distribution curve at distances around 2.8 Å, 5.6 Å, and 8.4 Å. The angle distribution curve exhibits two peaks, one broad peak around 90–110° and another around 160° in the case of scenarios A and B. These are the characteristics of the square-ice-like structure, as observed in previous studies.^36,45^ However, in the case of scenario C, the radial distribution curve and angle distribution curve showed single peaks, which indicates the presence of liquid water. The water present in this case has the typical tetragonal structure with a peak around 104° in the angle distribution curve.^36,45^ Lateral mean-square displacement (MSD) of water molecules was also obtained for all the scenarios, as shown in Fig. 3(c). The figure shows that the water molecules in the scenario C are more mobile when compared to other scenarios – thus indicating the higher proportion of liquid phase in the former. The lateral self-diffusion, parallel to the surface of the graphene sheet, for water molecules was obtained from these curves and compared with the self-diffusion coefficient of bulk liquid water at 300 K (from previous studies) as depicted in Fig. 3(d). The observed self-diffusion coefficient in scenarios A and B are much lower than the one for bulk liquid water (0.21 Å^2^ ps^−1^)^48^ and for scenario C it is nearly equal (0.22 Å^2^ ps^−1^). These observations indicate that in the case of scenario C, the water is present in a liquid state while in the other two scenarios, some ice-like structure is also present. Although both A and B structures contain ice-like properties, the self-diffusion coefficient in scenario B is lower. This is due to higher capillary forces experienced in A, where the thickness of the water film is lower than that in scenario B. This is in agreement with the observations made by Ghorbanfekr *et al.*^48^ where they showed that the diffusivity of water molecules increases when the thickness of the water film confined between hexagonal boron nitride nanocapillaries is reduced below 1 nm and it increases to the level of bulk liquid after increasing the thickness above 1 nm.

Typically, ice is found to possess hexagonal structure.^27,28^ However, in recent times, the square ice-like structure has been found both experimentally and theoretically, even at elevated temperatures, for water confined between two hydrophobic surfaces such as in graphene slits or bilayers.^27,32,45^ The driving force for the formation of a square-ice-like structure is the high lateral pressure (generally >1 GPa for the monolayer water film) created by the interaction forces between the confining surface and water atoms, which leads to enhanced hydrogen bonding within the water molecules.^49^ The lateral pressure on water molecules was also calculated in all the scenarios, as shown in Fig. 3(e). It can be observed that the lateral pressure in water molecules is greater than 1 GPa in all the cases. The lateral pressure in scenarios A and B is around 1.4 GPa and 1.7 GPa, respectively, which is sufficient for the evolution of square-ice-like patterns in water molecules at 300 K, in accordance with the phase diagram of the monolayer water film proposed by Kapil *et al.*^49^ In these cases, because of higher lateral pressure values, we observed patches of square ice in a very thin film of water even when one of the surfaces is weakly hydrophilic (*e.g.*, gold) as in scenarios A and B. However, the formation of ice or square ice is limited to a very thin film of water. At a higher thickness of water film, much higher lateral pressure is needed to reach ice-like phases as observed in the case of scenario C, where we do not observe ice-like structure even if the lateral pressure is higher, *i.e.* 1.8 GPa.^50^

**Fig. 4:**
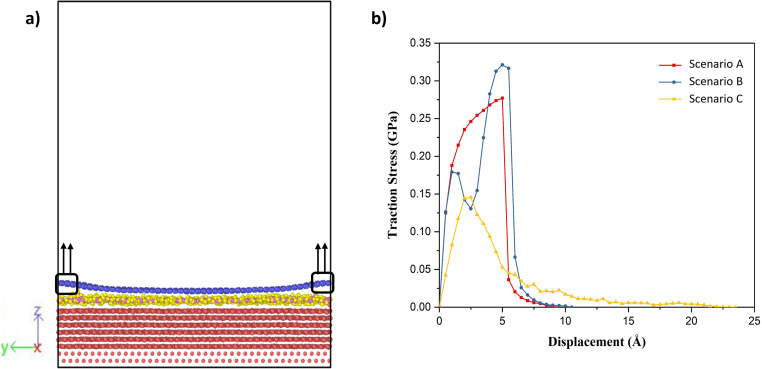
(a) Schematic of the traction–separation process during simulations, (b) calculated traction–separation curve for each of the scenarios.

Having established that variation of the phases of water with thickness in the nanoconfined region between gold and graphene, the next is to study their effect on the adhesion strength of graphene and gold interface. To study this aspect, we clamped a few carbon atoms of the graphene sheet along the edges in the *y*-direction and these were pulled in the *z*-direction, as shown in Fig. 4(a). Then, the total interaction force on graphene due to all other atoms was calculated at different separation/displacements in the *z*-direction of the graphene sheet from its initial position, as discussed in the Simulation methodology section below. Fig. 4(b) shows the calculated traction–separation graph for the three scenarios. A gradual increase in the traction stress is noted in both A and B scenarios until the displacements of less than 5 Å. For further displacement, we noted a steep fall in the stress and the stress declining to zero for displacement around 8–9 Å. The steep fall in the traction stress is a characteristic of the traction stress profile obtained while pulling graphene away from ice as observed in a previous study.^51^ This further proves the existence of an ice-like structure in scenarios A and B. We also observed a small peak around 2 Å in scenario B, after which the stress decreased and then again increased with further displacement, possibly due to the movement of wrinkles in graphene from the centre of the box to the edge of the box as explained later. The traction–separation profile for scenario C was found to be similar to the traction separation curve when graphene was pulled away from liquid water, as observed in a previous study.^16^ In this case, the stress increases until the displacement of 2.5 Å, after which it decreases exponentially. The reason behind the sudden fall in traction stress in scenarios A and B and the slow gradual fall in the case of scenario C is the sudden detachment of the graphene sheet from the water film in A and B (somewhere between the displacement of 5 Å and 6 Å) and the slow detachment of graphene and adherence to the liquid water in some regions in the case of scenario C (until a displacement of 22 Å) as shown in ESI Video V1.† Interestingly, it can be also observed from this figure that the presence of ice structure in scenarios A and B, leads to short-ranged (less than 1.5 nm) interaction between gold and graphene. On the other hand, in scenario C, the interaction is long-ranged (more than 3 nm) due to the presence of liquid water. It is also evident from this graph that the maximum traction stress required to pull the graphene sheet is higher in A and B when compared with C, which means the adhesion strength of the graphene sheet with an ice-like water structure and the gold substrate is higher than in comparison to the scenario when liquid water is present between gold and graphene.

**Fig. 5:**
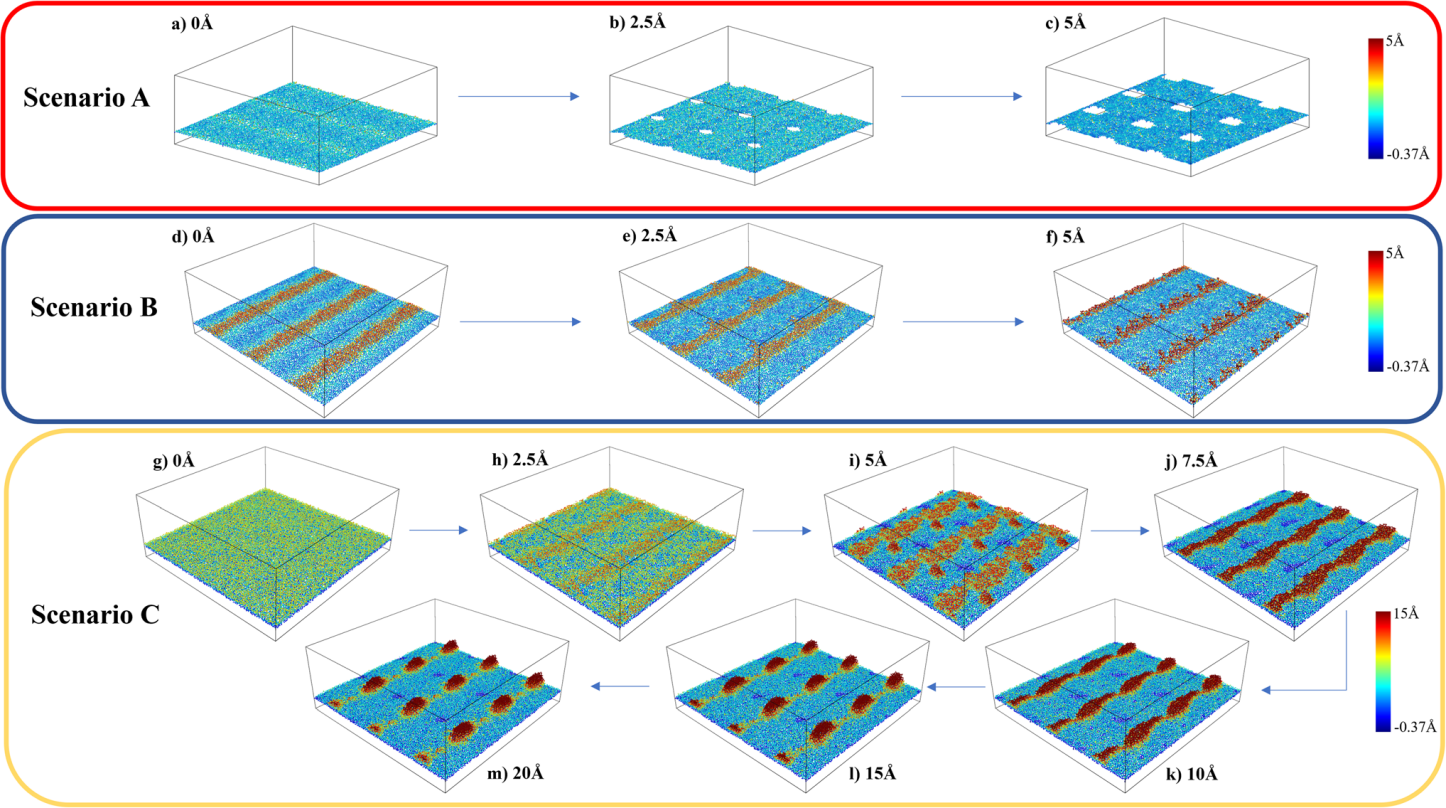
Snapshots of water molecules (3 × 3 periodic replication) from MD simulations during the traction–separation process (atoms of gold and graphene are omitted for better understanding) with colour coding reflecting the height in the *z*-direction.

The variations in the local structure of water molecules during the traction separation process can explain the detachment mechanism of graphene from the water–gold interface. To this end, we observed the morphology evolution in the water film during the traction separation process in all the scenarios, as shown in Fig. 5 and ESI Video 1.† This figure shows the 3 × 3 periodic replication snapshots after equilibrium and during the traction process of the water film trapped between gold and graphene with colour coding according to the height contour of the water molecules. From Fig. 5 (a–c), it can be observed that in the case of scenario A the new cavities are formed in the water film during the traction process. The size of these cavities increased with traction until 5 Å and got stabilised after this separation distance. This means that there was no adhesion between the graphene sheet and water beyond this separation and thus, a steep fall in the traction separation curve was observed at this separation distance in the case of scenario A, as shown in Fig. 4(b). In the case of scenario B, from Fig. 5(d–f), no pores or cavities were observed, and the capillary bridges (red coloured patches) formed due to the bilayer water structure were seen after the equilibration process. During the traction separation process, these capillary bridges move from centre towards the edge of the box and a slight change in the height of these bridges was noticed. After a separation of 5–6 Å, the heights of the capillary bridge got stabilised, which again shows that there was no interaction between graphene and water beyond this separation distance. Thus, a steep decline in the traction separation graph in the scenario B is explained as well. In the case of C, initially, there is a smooth water film found between gold and graphene after equilibration observed in Fig. 5(g). During the traction–separation process, the capillary bridges were formed initially at lower displacements, which broke into a conical structure at higher displacements, as observed from Fig. 5(h–m). The transformation of uniform water film from the capillary bridge to a conical structure due to the presence of liquid water was also found in previous theoretical work^16^ on the traction–separation process of graphene over a wet-silica substrate. This shows the long-range interaction between gold and liquid water until the separation of 15 Å in the case of scenario C. From the above discussion, it is evident that the morphologies of the entrapped water film, with different heights and phases, during the traction–separation process are not similar. Instead, they are quite different in nature.

## Conclusion

Molecular dynamics simulations employed to study the structure and traction–separation properties of entrapped confined water molecules between gold and graphene suggest the presence of an ice-like structure in thinner water films (<5 Å) and liquid water structure at higher thickness of water films. This work also predicts the formation of patches of square-ice-like structure between a hydrophobic and a hydrophilic surface at very low separation (<5 Å). Due to the presence of different phases of water, the traction separation study highlights the differences in adhesion strength of graphene with the heights of water film. The varying structures of water could lead to different band gaps^52^ and hence, the structure of water nanoconfined in the nanoregions between gold and graphene could influence the electronic properties of graphene devices such as GFETs. This will be studied in future work with the fabrication of GFETs. The results presented in this work are expected to help experimentalists understand the influence of microscopic pictures of water films confined in wet-transferred graphene devices.

## Simulation methodology

A simulation box of size 10 nm × 10 nm × 50 nm was used for all the simulations presented in this work. It consisted of a 2.5 nm thick Au (111) ([111] being the *z*-direction) substrate at the bottom of the box in which a few of the bottom layers (0.6 nm thick) were kept fixed while others were free to move. A graphene sheet of size 10 nm × 10 nm was placed over this gold substrate. Water molecules were added between the gold and graphene using PACKMOL.^53^ Periodic boundary conditions were applied in all the dimensions. A vacuum was kept in the *z*-direction above the graphene layer to avoid any interaction with its periodic image.

The Au (111) substrate was modelled using the Embedded Atom Method (EAM) forcefield parameters^54,55^ while Adaptive Intermolecular Reactive empirical Bond Order (AIREBO)^56^ parameters were used for the graphene layer. The SPC/E forcefield parameters^57^ were used to model the water molecules. The charges on gold and graphene atoms were taken as zero while for O and H atoms of water molecules, the charges were −0.8476 e and 0.4238 e, respectively. The interaction between gold and graphene, graphene–water and gold–water was modelled using the 6, 12 LJ potential with eqn (1), and these parameters are given in ESI Table S1.†1
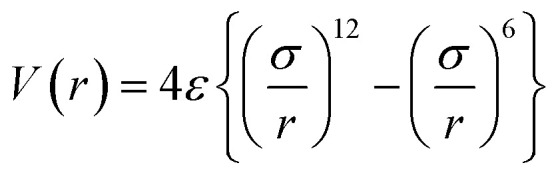


To measure the equilibrium distance between the Au (111) and graphene, a similar box was used without water molecules. This box was subjected to energy minimisation at 0 K followed by NVT ensemble equilibration at 300 K for 500 ps.

The simulation box with confined water molecules was also energy minimised and then equilibrated using NVT ensemble at 300 K for 500 ps. Water structures formed during the last 50 ps were used at every 1 ps and analysed to obtain the average lateral O–O radial distribution and O–O–O angle distribution curves. Later, the equilibrated systems were used to obtain the traction–separation curves.

During the separation process, the sides of the graphene sheet were clamped and a series of displacements of 0.5 Å was applied stepwise in the positive *z* direction to these clamped graphene atoms. Between every step of the displacement of atoms, a relaxation process was applied in the NVT ensemble for 100 ps. At each separation displacement, the total interaction force on graphene due to all other atoms (Au, O and H atoms) was computed using the following function:2
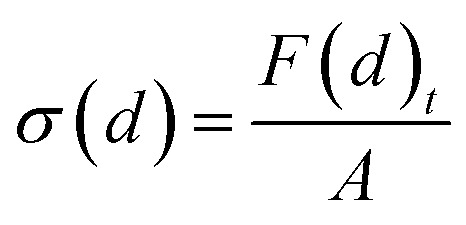
where *A* is the area of the undeformed graphene sheet, *d* is the applied displacement on graphene atoms and *F*(*d*)_*t*_ is time averaged total force on the graphene sheet as a function of *d*. A similar method and equation were used successfully by Gao^16^ to obtain the traction–separation curve for the silica–water–graphene system.

Lateral MSD for water in the direction parallel to the surface of the graphene sheet was calculated and using it, the self-diffusion coefficient, *D*_self_ for water molecules was also calculated by using the following equation:^48^3
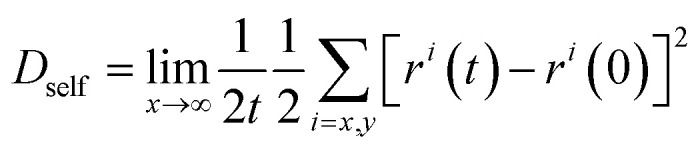
where *t* is time, *r*^*i*^(*t*) refers to the position of an atom at time *t* and *r*^*i*^(0) refers to its initial position.

All simulations were run in the LAMMPS^58^ simulation package. The timestep used for the entire simulation was 1 fs. OVITO^59^ visualisation software was used to analyse the simulations.

## Conflicts of interest

The authors declare no conflict of interest.

## Supplementary Material

NA-006-D3NA00878A-s001

NA-006-D3NA00878A-s002
