# Supplementary material for: Molecular dynamics simulation-based study to analyse the properties of entrapped water between gold and graphene 2D interfaces
Source: Nanoscale Adv. 2024 Mar 21;6(9):2371–9. doi: 10.1039/d3na00878a (PMC11059550; doi:10.1039/d3na00878a)
Supplement: NA-006-D3NA00878A-s001 [file NA-006-D3NA00878A-s001.pdf]

Electronic Supplementary Material (ESI) for Nanoscale Advances.
This journal is © The Royal Society of Chemistry 2024

Supporting Information

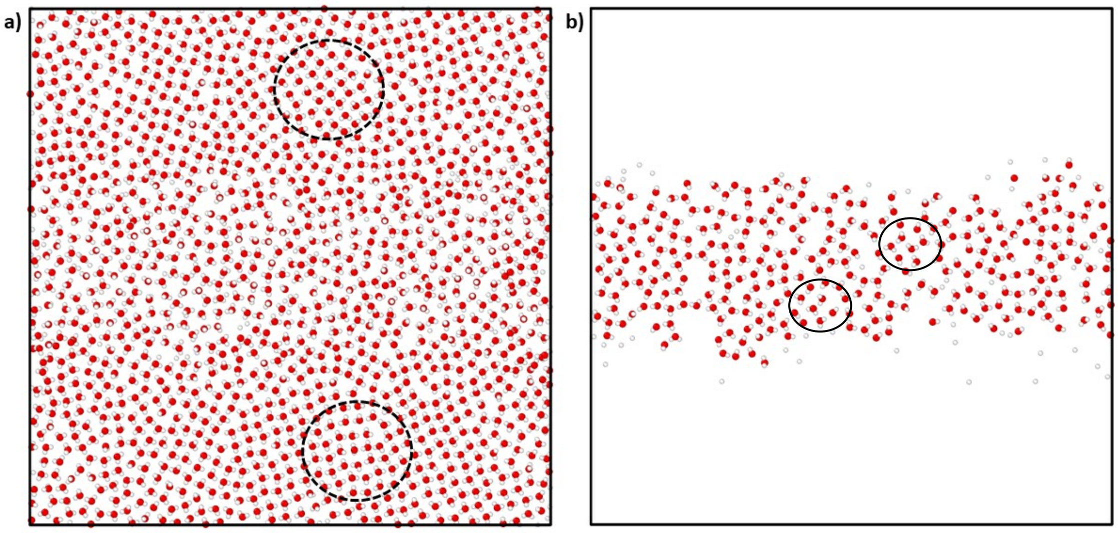

Figure S1: Top view of the snapshots of (a) bottom layer and (b) top layer seen from top in the case of scenario B after equilibration. The black dashed circles show the presence of square ice pattern in these water structures.

Table S1: LJ potential coefficients used for various pair of atoms used in the simulations *(* denotes any atom type out of Au, C, O and H)*

Atom Pairs	ε (meV)	σ (Å)
Au - C	8	3.42
Au – O	25.5	3.6
C - O	6.74	3.17
O - O	4.06	3.19
H - *	0	0
